# Angle-resolved AlN/SiO_2_ phoxonic microcavity for optical–acoustic readout of sweat cortisol perturbations toward future receptor-mediated chemical transduction

**DOI:** 10.1039/d6ra06162a

**Published:** 2026-09-21

**Authors:** Arafa H. Aly

**Affiliations:** a TH-PPM Group, Department of Physics, Faculty of Science, Beni-Suef University Beni Suef 62511 Egypt arafaaly@aucegypt.edu arafa.hussien@science.bsu.edu.eg

## Abstract

Sweat cortisol is a chemically relevant biomarker associated with physiological stress responses, but its direct optical and acoustic perturbation in aqueous media is extremely weak. Here, we present a numerical design of an AlN/SiO_2_ phoxonic microcavity framework toward future receptor-mediated chemical transduction, based on alternating aluminium nitride and silicon dioxide layers and considering a cortisol-responsive aptagel layer as a prospective receptor-mediated transduction element. The structure was analysed using angle-resolved optical transfer-matrix modelling for TE and TM polarizations together with longitudinal acoustic transfer-matrix calculations. Direct bulk-cortisol modelling, governed by Langmuir binding with KD = 500 nM, produced a refractive-index perturbation of 9.802 × 10^−9^ RIU at 50 ng mL^−1^, corresponding to an optical wavelength shift of 0.01153 pm at normal incidence. Geometry optimization increased the optical readout response to 0.02711 pm at 75° incidence using a defect-thickness factor of 1.60, providing a 1.77-fold optical readout enhancement. Acoustic modelling identified a loaded defect resonance at 6.158 GHz within a Bloch stop band of 5.952–9.644 GHz. After numerical convergence validation, the optimized acoustic mirror configuration with *N* = 12 yielded a quality factor of 1.99 × 10^5^, an interpolated FWHM of 0.0309 MHz, and an 82.51-fold acoustic readout-quality enhancement. These improvements represent enhanced optical–acoustic resonance tracking rather than biochemical amplification. The proposed AlN/SiO_2_ phoxonic microcavity therefore provides a physics-based readout framework for cortisol-containing sweat media, while practical stress-associated sensing requires experimentally calibrated receptor-mediated transduction.

## Abstract 2

Conceptual design of an angle-resolved AlN/SiO_2_ phoxonic microcavity enabling enhanced optical–acoustic readout of sweat cortisol-related perturbations toward future receptor-mediated chemical transduction.

## Graphical abstract

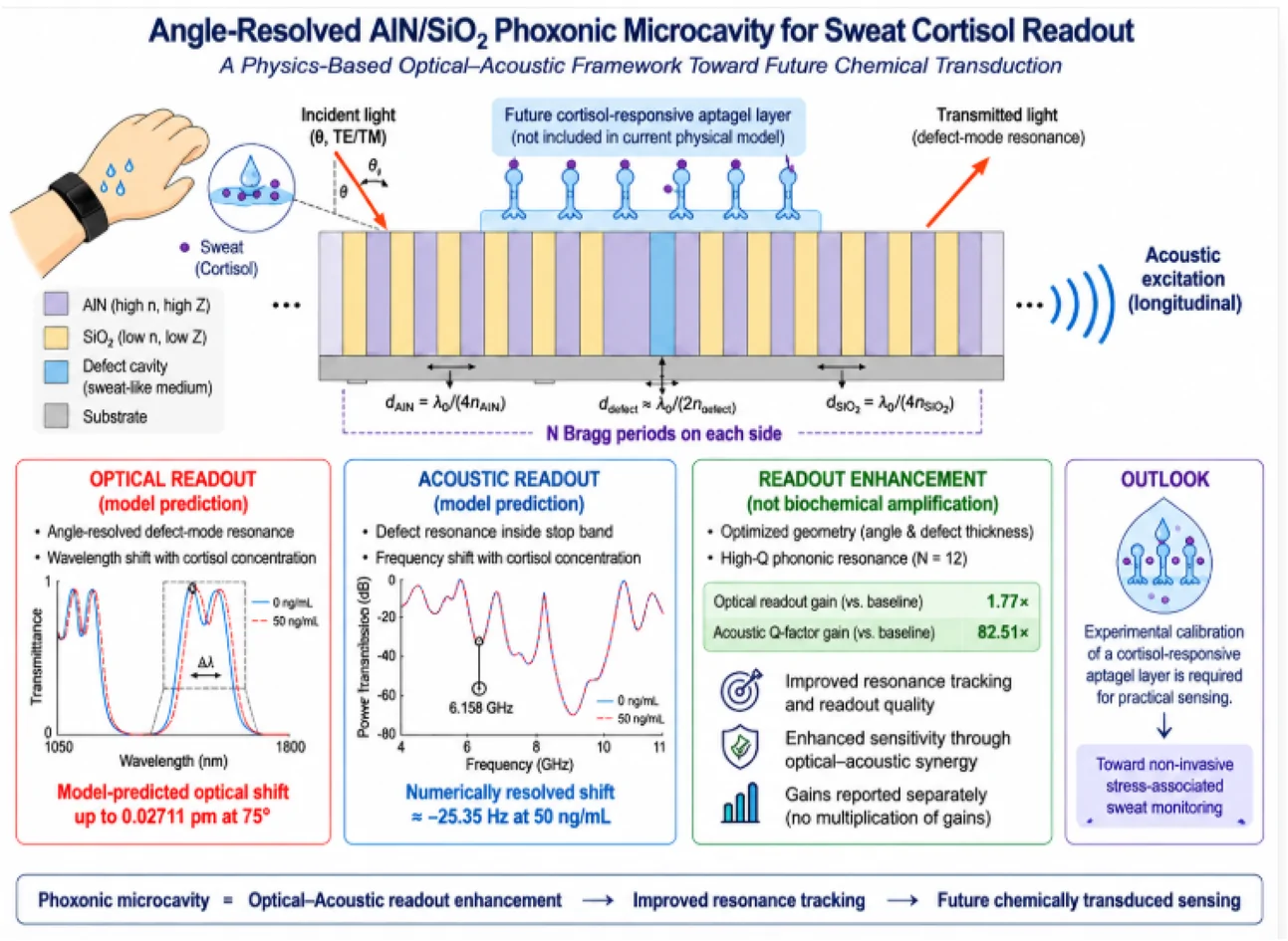

## Introduction

Sweat is increasingly considered a chemically informative biofluid for non-invasive monitoring because it contains small molecules, ions, metabolites, and hormones that can reflect changes in physiological state. Among these biomarkers, cortisol has received particular attention because it is associated with hypothalamic–pituitary–adrenal axis activity and stress-associated endocrine responses. However, cortisol is a small steroid molecule, and its direct effect on the refractive index, density, and acoustic properties of aqueous sweat-like media is intrinsically weak. This creates a major sensing challenge: although cortisol is chemically relevant, the direct optical or acoustic perturbation produced by its bulk concentration is often insufficient for robust detection without a selective transduction mechanism. Recent wearable aptameric, electrochemical, field-effect transistor, and capacitive platforms have demonstrated the practical interest in cortisol monitoring, while also highlighting the continuing need for improved selectivity, stability, and signal-transduction strategies in complex biofluids.^1–6^

Beyond cortisol-specific sensing, recent progress in non-invasive chemical sensing has expanded toward broader biomarker analysis, portable chemical detection, and multimodal sensing platforms. Body-fluid-based sensing approaches have demonstrated the importance of molecular recognition strategies for extracting physiological information from complex biological environments. In addition, portable volatilomics technologies and advanced chemical-sensing materials have provided new opportunities for rapid and non-invasive monitoring through analysis of complex chemical signatures. Integrated sensing platforms that combine chemical recognition with physical signal transduction further demonstrate the potential of multimodal architectures for improving sensing reliability and expanding biomedical applications.^7–11^ These developments emphasize that future non-invasive sensing systems require not only selective chemical interaction but also stable and sensitive physical readout mechanisms capable of converting weak molecular events into measurable signals. Compared with existing chemical and wearable sensing platforms that primarily rely on molecular recognition and electrical or optical signal conversion, the present work explores a complementary phoxonic strategy in which optical and acoustic resonance engineering is used to enhance the readout of weak biochemical perturbations.

Photonic and phoxonic crystals provide an attractive route for enhancing readout quality because localized defect modes can convert small chemical or mechanical variations inside a cavity into measurable shifts in resonance wavelength, resonance frequency, linewidth, and quality factor.^12^ In one-dimensional defective photonic crystals, the introduction of a central cavity breaks the periodic symmetry and produces a sharp resonance within the photonic band gap. This concept has been widely explored for chemical sensing, organic-compound detection, cancer-related refractive-index sensing, hormone detection, and biomedical fluid analysis. Recent studies have shown that defect-engineered multilayer photonic structures can be optimized through material selection, phase-change integration, graphene incorporation, and cavity engineering, confirming the value of compact multilayer architectures for chemically driven optical sensing.^13–16^

Despite this progress, most photonic cortisol-sensing concepts rely mainly on optical resonance shifts. This leaves an important research gap: weak cortisol-induced perturbations may be more reliably evaluated when optical and acoustic resonances are considered as complementary readout channels within the same structured platform. Phononic crystal sensors are particularly useful in this context because acoustic defect modes are sensitive to variations in density, sound velocity, compressibility, and viscosity of the defect medium. Recent developments in phononic crystal sensing, liquid-loaded acoustic structures, Fano-resonant phononic systems, and coupled-cavity engineering have demonstrated that acoustic resonance quality can be improved through appropriate mirror-period selection and defect engineering.^17–28^

The present work addresses this gap by proposing an angle-resolved AlN/SiO_2_ phoxonic microcavity for the optical–acoustic readout of cortisol-containing sweat media. Aluminium nitride and silicon dioxide are selected as alternating multilayer materials because they provide useful optical and acoustic contrast while remaining compatible with thin-film and micro-device design. The central defect region is treated as a sweat-like cavity that can later be functionalized with a cortisol-responsive aptagel layer. The aim is not to claim direct psychiatric diagnosis, nor to overstate the bulk response of cortisol. Instead, the study distinguishes between three effects: the direct bulk-cortisol perturbation, the optical–acoustic readout enhancement produced by geometry and mirror optimization, and the future biochemical transduction expected from an experimentally calibrated aptagel layer.

Using transfer matrix calculations, the work evaluates TE/TM optical responses under oblique incidence together with longitudinal acoustic defect resonances. The results show that direct bulk cortisol produces only a very small refractive-index perturbation and a weak acoustic frequency shift. However, angle and defect-thickness optimization improve the optical readout, while increasing the number of acoustic Bragg mirror periods enhances the validated acoustic quality factor. This establishes the proposed AlN/SiO_2_ phoxonic microcavity as a readout-quality-enhanced platform for future chemically transduced sweat cortisol sensing, while maintaining a clear distinction between physical readout enhancement and biochemical amplification.

### Physical and mathematical framework of the model

This section summarizes only the physical and mathematical relations explicitly implemented in the MATLAB model. The formulation represents a one-dimensional AlN/SiO_2_ phoxonic microcavity with a central sweat-like defect layer. The defect is treated either as a direct dilute cortisol-in-water medium or, when enabled in the code, as an experimentally calibrated aptagel layer. The current model output corresponds to the direct bulk-cortisol physical-control mode.

### Theoretical model and system configuration

The cortisol mass concentration was converted to molar concentration before evaluating the receptor-occupancy response:1
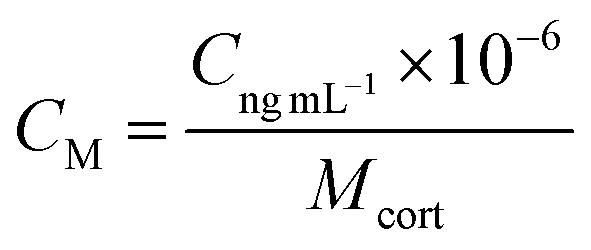
where *C*_M_ is the cortisol molar concentration in mol L^−1^, *C*_ng mL^−1^_ is the cortisol mass concentration in ng mL^−1^, and *M*_cort_ is the molar mass of cortisol in g mol^−1^.

The saturation response of the cortisol-binding sites was evaluated using a Langmuir occupancy relation:2
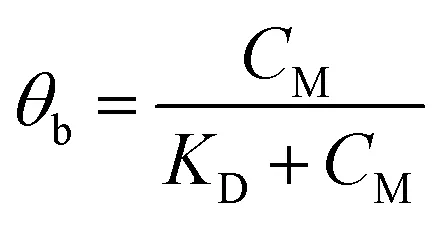
where *θ*_b_ is the fractional binding occupancy, *C*_M_ is the cortisol molar concentration, and *K*_D_ is the cortisol-aptamer dissociation constant used in the code.

For the direct-bulk control mode, the dilute cortisol volume fraction was estimated from the mass concentration and the solid cortisol density:3
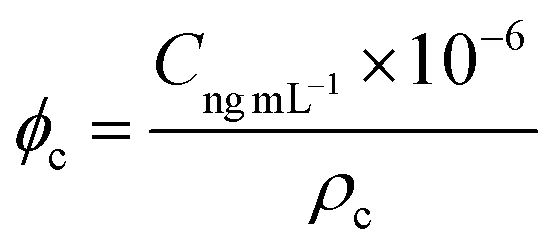
where *ϕ*_c_ is the dilute cortisol volume fraction, *C*_ng mL^−1^_ is the cortisol mass concentration, and *ρ*_c_ is the density assigned to solid cortisol in kg m^−3^.

The direct refractive-index perturbation was calculated using the Lorentz–Lorenz mixing rule:4
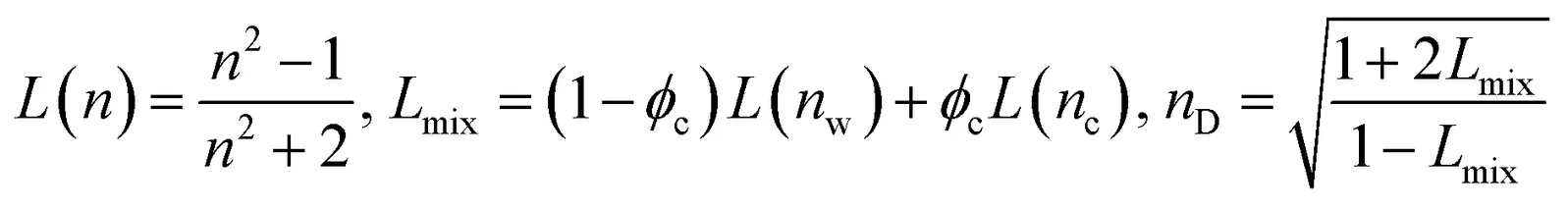
where *L*(*n*) is the Lorentz–Lorenz function, *L*_mix_ is the mixed Lorentz–Lorenz term, *ϕ*_c_ is the cortisol volume fraction, *n*_w_ is the refractive index of water at the modelling wavelength, *n*_c_ is the refractive index assigned to solid cortisol, and *n*_D_ is the effective refractive index of the defect layer.

The defect density and sound speed were calculated under the fixed-water-bulk-modulus assumption:5
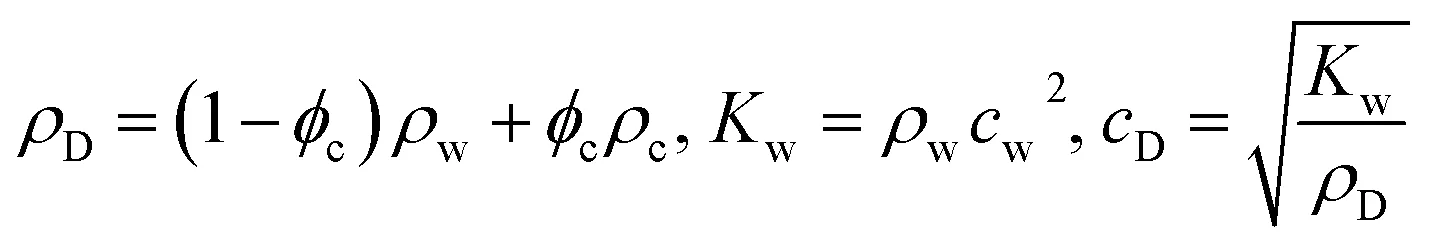
where *ρ*_D_ is the defect density, *ρ*_w_ is the water density, *ρ*_c_ is the solid cortisol density, *K*_w_ is the effective bulk modulus of water, *c*_w_ is the longitudinal sound speed in water, and *c*_D_ is the defect sound speed.

The SiO_2_ refractive index was evaluated using the wavelength-dependent Sellmeier expression implemented in the code:6
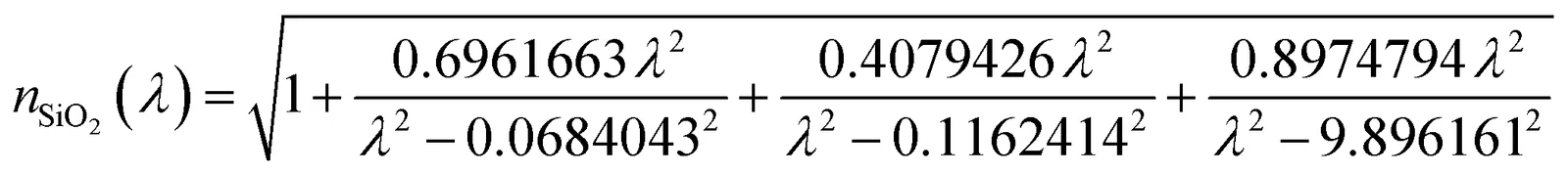
where *n*_SiO_2__(*λ*) is the wavelength-dependent refractive index of silica and *λ* is the optical wavelength in µm, as used in the code.

The starting Bragg-stack geometry was defined by a quarter-wave optical design and a half-wave defect cavity at the central wavelength:7
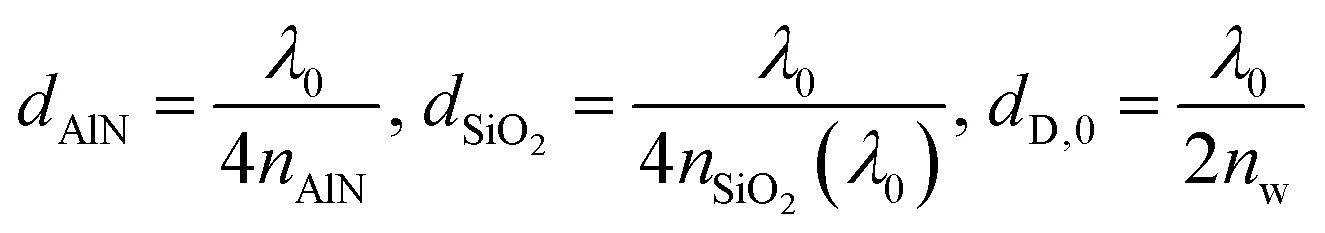
where *d*_AlN_ is the AlN layer thickness, *d*_SiO_2__ is the SiO_2_ layer thickness, *d*_D,0_ is the initial defect thickness, *λ*_0_ is the design wavelength, *n*_AlN_ is the AlN refractive index, *n*_SiO_2__(*λ*_0_) is the silica refractive index at *λ*_0_, and *n*_w_ is the water refractive index.

### Transfer-matrix formulation

The optical propagation angle inside each layer was calculated from Snell's law:8
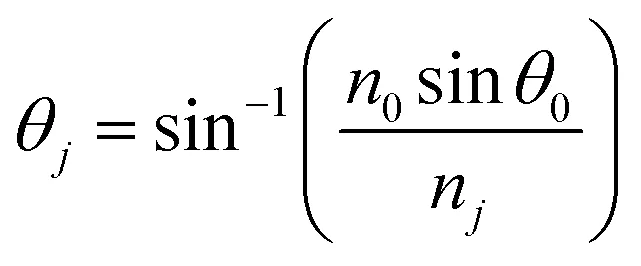
where *θ*_*j*_ is the propagation angle inside layer *j*, *θ*_0_ is the incidence angle in air, *n*_0_ is the refractive index of the incident medium, and *n*_*j*_ is the refractive index of layer *j*.

For each optical layer, the phase thickness and TE/TM admittances were implemented as:9
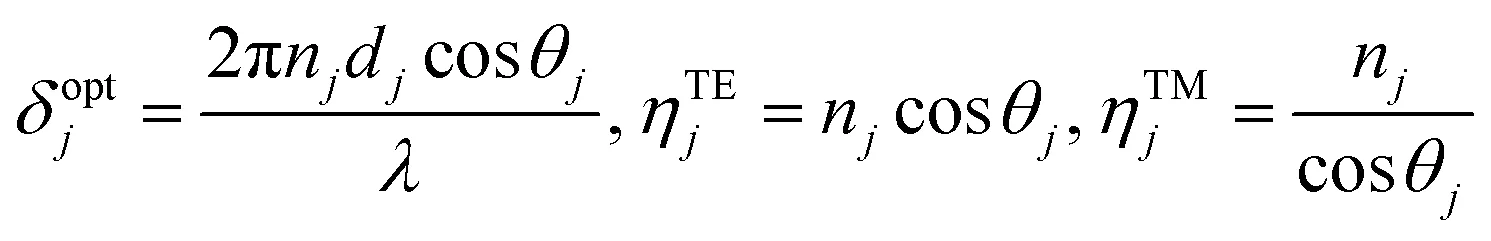
where *δ*^opt^_*j*_ is the optical phase thickness, *n*_*j*_ is the refractive index, *d*_*j*_ is the physical thickness, *θ*_*j*_ is the internal propagation angle, *λ* is the wavelength, and *η*^TE^_*j*_ and *η*^TM^_*j*_ are the layer admittances for TE and TM polarization.

The optical characteristic matrix of a single layer was then written as:10
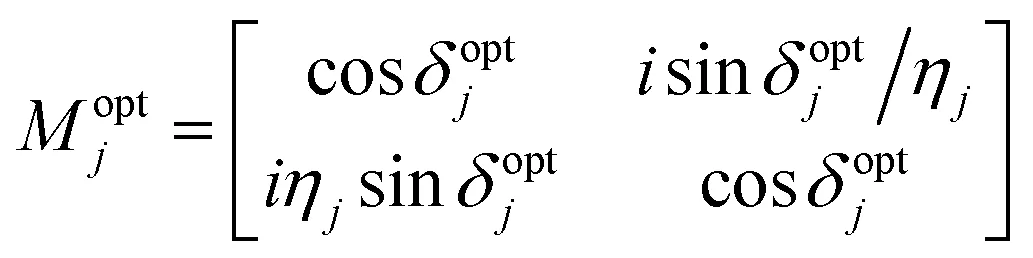
where *M*^opt^_*j*_ is the optical layer matrix, *δ*^opt^_*j*_ is the optical phase thickness, and *η*_*j*_ is the TE or TM admittance selected for the polarization being calculated.

The full optical stack was assembled in the same left-defect-right sequence as the MATLAB loops:11
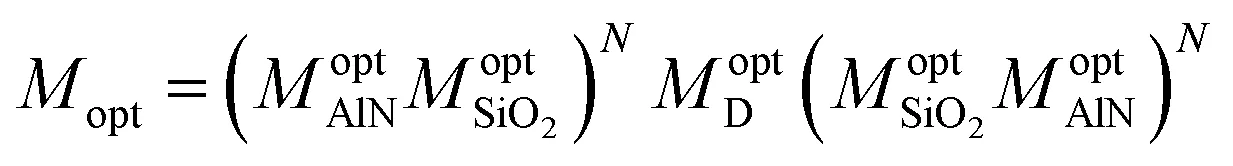
where *M*_opt_ is the total optical transfer matrix, *M*^opt^_AlN_ is the AlN layer matrix, 
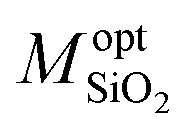
 is the SiO_2_ layer matrix, *M*^opt^_D_ is the defect-layer matrix, and *N* is the number of Bragg periods in each mirror.

### Optical response formulation

The optical amplitude transmission and power transmittance were computed from the total matrix as:12
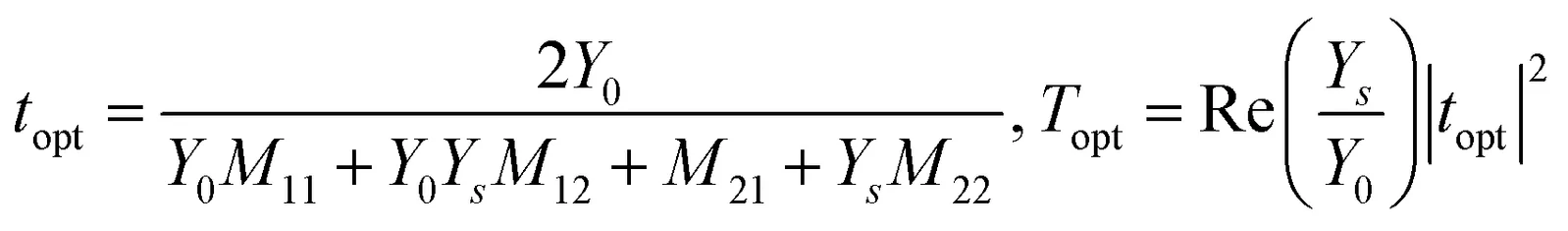
where *t*_opt_ is the optical amplitude transmission coefficient, *T*_opt_ is the optical power transmittance, *Y*_0_ is the optical admittance of the incident medium, *Y*_*s*_ is the optical admittance of the substrate medium, and *M*_11_, *M*_12_, *M*_21_, and *M*_22_ are the elements of *M*_opt_.

The angle-dependent cavity estimate used for sensitivity mapping was:13
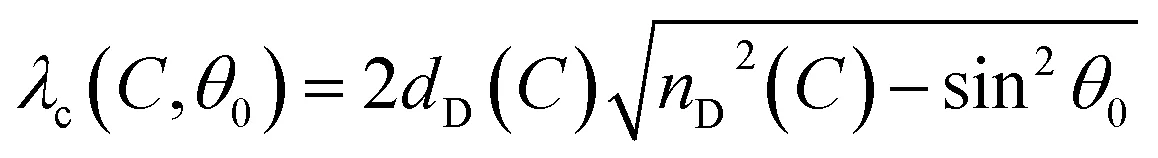
where *λ*_c_ is the cavity-estimated resonance wavelength, *C* is the cortisol concentration, *θ*_0_ is the incidence angle in air, *d*_D_(*C*) is the defect thickness, and *n*_D_(*C*) is the defect refractive index at concentration *C*.

### Acoustic response formulation

The acoustic impedance and phase thickness of each layer were computed as:14
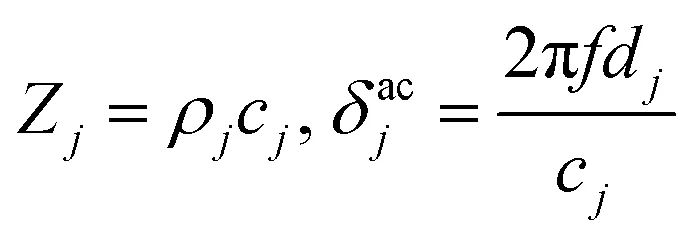
where *Z*_*j*_ is the longitudinal acoustic impedance, *ρ*_*j*_ is the density, *c*_*j*_ is the longitudinal sound speed, *δ*^ac^_*j*_ is the acoustic phase thickness, *f* is the acoustic frequency, and *d*_*j*_ is the layer thickness.

The acoustic transfer matrix of each layer was implemented as:15
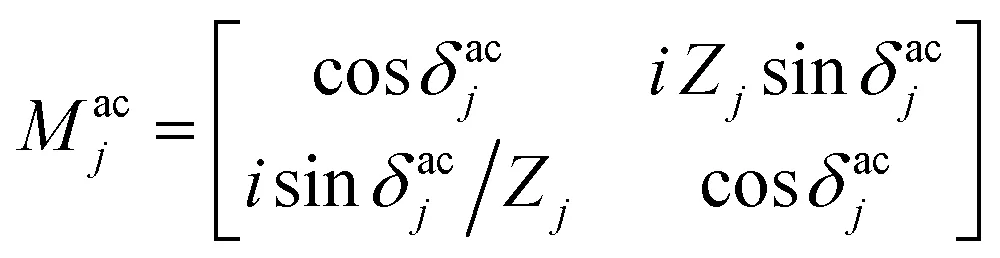
where *M*^ac^_*j*_ is the acoustic layer matrix, *δ*^ac^_*j*_ is the acoustic phase thickness, and *Z*_*j*_ is the acoustic impedance of layer *j*.

The acoustic Bragg phase center and Bloch stop-band condition were evaluated as:16
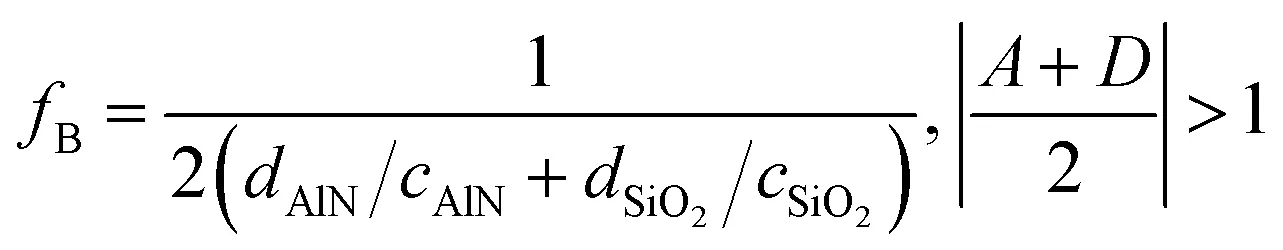
where *f*_B_ is the acoustic Bragg phase-center frequency, *d*_AlN_ and *d*_SiO_2__ are the layer thicknesses, *c*_AlN_ and *c*_SiO_2__ are the longitudinal sound speeds, and *A* and *D* are the diagonal elements of the single-cell acoustic matrix *M*_cell_.

The acoustic amplitude transmission and power transmittance were calculated as:17
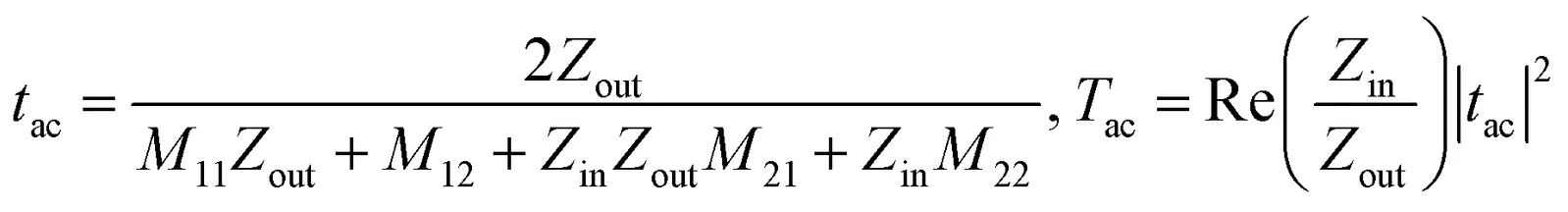
where *t*_ac_ is the acoustic amplitude transmission coefficient, *T*_ac_ is the acoustic power transmittance, *Z*_in_ is the acoustic impedance of the input medium, *Z*_out_ is the acoustic impedance of the output medium, and *M*_11_, *M*_12_, *M*_21_, and *M*_22_ are the elements of the full acoustic transfer matrix.

The unloaded liquid-defect acoustic estimate was used only as a reference calculation:18
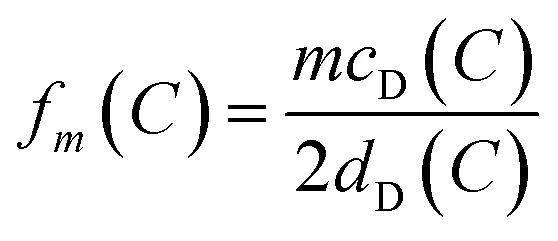
where *f*_*m*_ is the unloaded liquid-cavity frequency estimate, *m* is the selected acoustic mode order, *c*_D_(*C*) is the defect sound speed, and *d*_D_(*C*) is the defect thickness.

### Perturbation or sensing model

The optical and acoustic shifts were calculated relative to the zero-cortisol baseline:

19 Δ*λ*(*C*,*θ*_0_) = 1000[*λ*_c_(*C*,*θ*_0_) − *λ*_c_(0,*θ*_0_)], Δ*f*(*C*) = *f*(*C*) − *f*(0)

where Δ*λ* is the wavelength shift in pm, *λ*_c_ is expressed in nm, *θ*_0_ is the incidence angle, Δ*f* is the frequency shift in Hz, and *f*(*C*) and *f*(0) are the concentration-dependent and baseline resonance frequencies.

The optical sensitivity and geometry-optimization gain were evaluated as:20
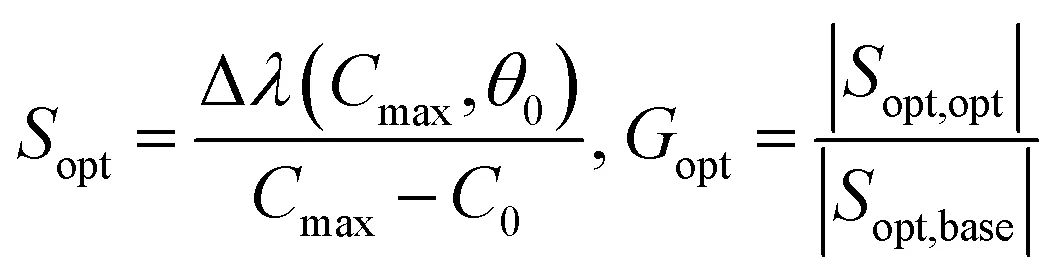
where *S*_opt_ is the optical sensitivity in pm (ng mL^−1^)^−1^, *C*_max_ is the largest cortisol concentration used in the sweep, *C*_0_ is the baseline concentration, *G*_opt_ is the geometry-based optical gain, *S*_opt,opt_ is the optimized sensitivity, and *S*_opt,base_ is the baseline best-angle sensitivity.

### Resonance shift and sensitivity analysis

Sub-grid acoustic peak positions were obtained by fitting a local scaled quadratic around the grid maximum:21
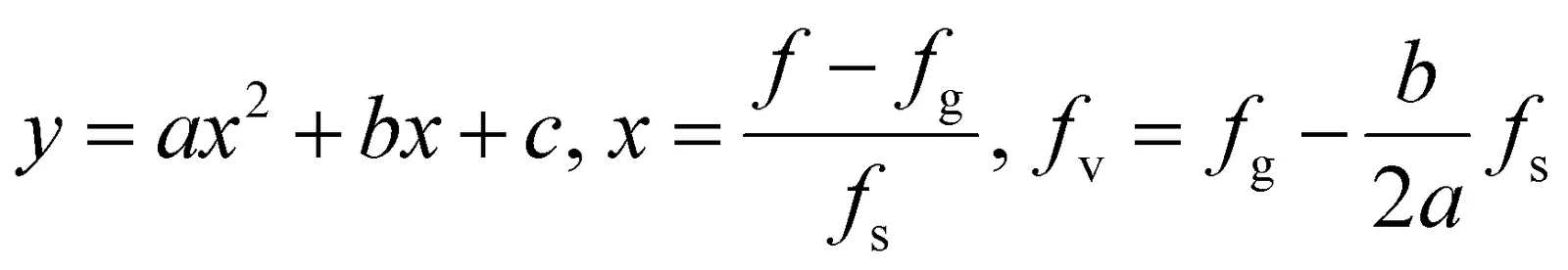
where *y* is the local acoustic transmittance, *a*, *b*, and *c* are least-squares quadratic coefficients, *x* is the centred and scaled local frequency coordinate, *f* is frequency, *f*_g_ is the grid-maximum frequency, *f*_s_ is the local scaling frequency, and *f*_v_ is the sub-grid vertex frequency.

### Quality factor and detection-limit metrics

The resonance linewidth and quality factor were calculated from the half-power crossings:22
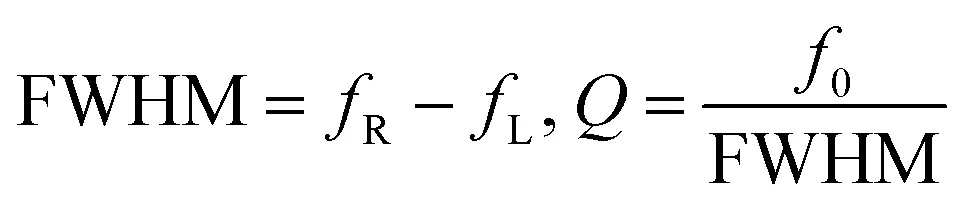
where FWHM is the full width at half maximum, *f*_L_ and *f*_R_ are the left and right half-power crossing frequencies, *Q* is the quality factor, and *f*_0_ is the selected resonance frequency.

The code defines experimental detectability only when measured peak-fit uncertainty is supplied:23
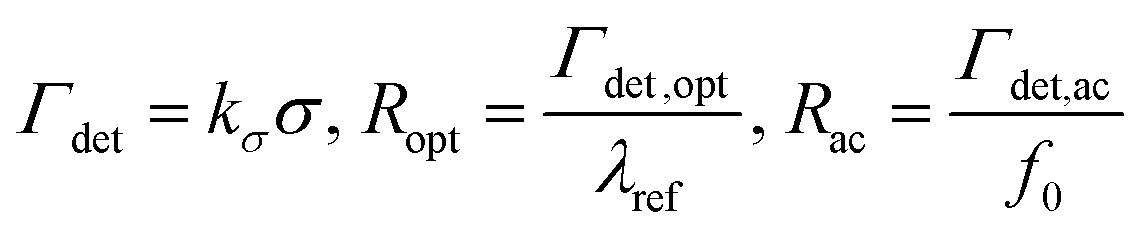
where *Γ*_det_ is the detection threshold, *k*_*σ*_ is the detection-sigma multiplier used in the code, *σ* is the measured peak-fit standard deviation, *R*_opt_ is the optical required fractional phase change, *Γ*_det,opt_ is the optical detection threshold, *λ*_ref_ is the reference cavity wavelength, *R*_ac_ is the acoustic required fractional phase change, and *Γ*_det,ac_ is the acoustic detection threshold.

The optical geometry gain and the acoustic quality-factor enhancement are reported separately because they originate from different physical mechanisms and cannot be combined into a single sensing metric without an experimentally validated uncertainty model.

## Numerical implementation

The convergence of the acoustic shift and the validated high-*Q* result was assessed by the percent change between successive frequency-step refinements:24
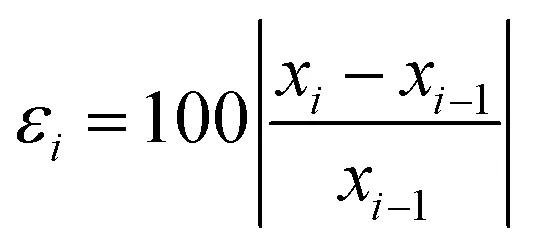
where *ε*_*i*_ is the percent change at refinement step *i*, *x*_*i*_ is the current numerical quantity being tested, and *x*_*i*−1_ is its value at the previous frequency-step refinement. In the code, *x*_*i*_ is applied to the sub-grid frequency shift, the validated quality factor, or the interpolated FWHM.

The optical transfer-matrix calculations were performed over a wavelength range of 1050–1800 nm with an initial spectral step of 0.25 nm. Optical resonance verification was subsequently performed using refined wavelength grids down to 0.01 pm, and the resonance wavelength was extracted using a local five-point quadratic fitting procedure around the maximum transmission peak.

The acoustic transfer-matrix calculations were initially evaluated over a frequency range of 4–11 GHz with a frequency step of 0.002 GHz. The selected defect resonance was further refined using frequency steps of 50 kHz and additional convergence refinement down to 1 kHz for the final quality-factor validation. Acoustic peak extraction was performed using local quadratic interpolation, and the linewidth was obtained from half-power crossing points.

## Results and discussion

Fig. 1 illustrates the proposed angle-resolved AlN/SiO_2_ phoxonic microcavity architecture for optical–acoustic readout of sweat cortisol-related perturbations. The structure combines optical defect-mode interrogation with acoustic defect resonance tracking, while the future cortisol-responsive aptagel layer is considered as a prospective transduction element requiring experimental calibration.

**Fig. 1:**
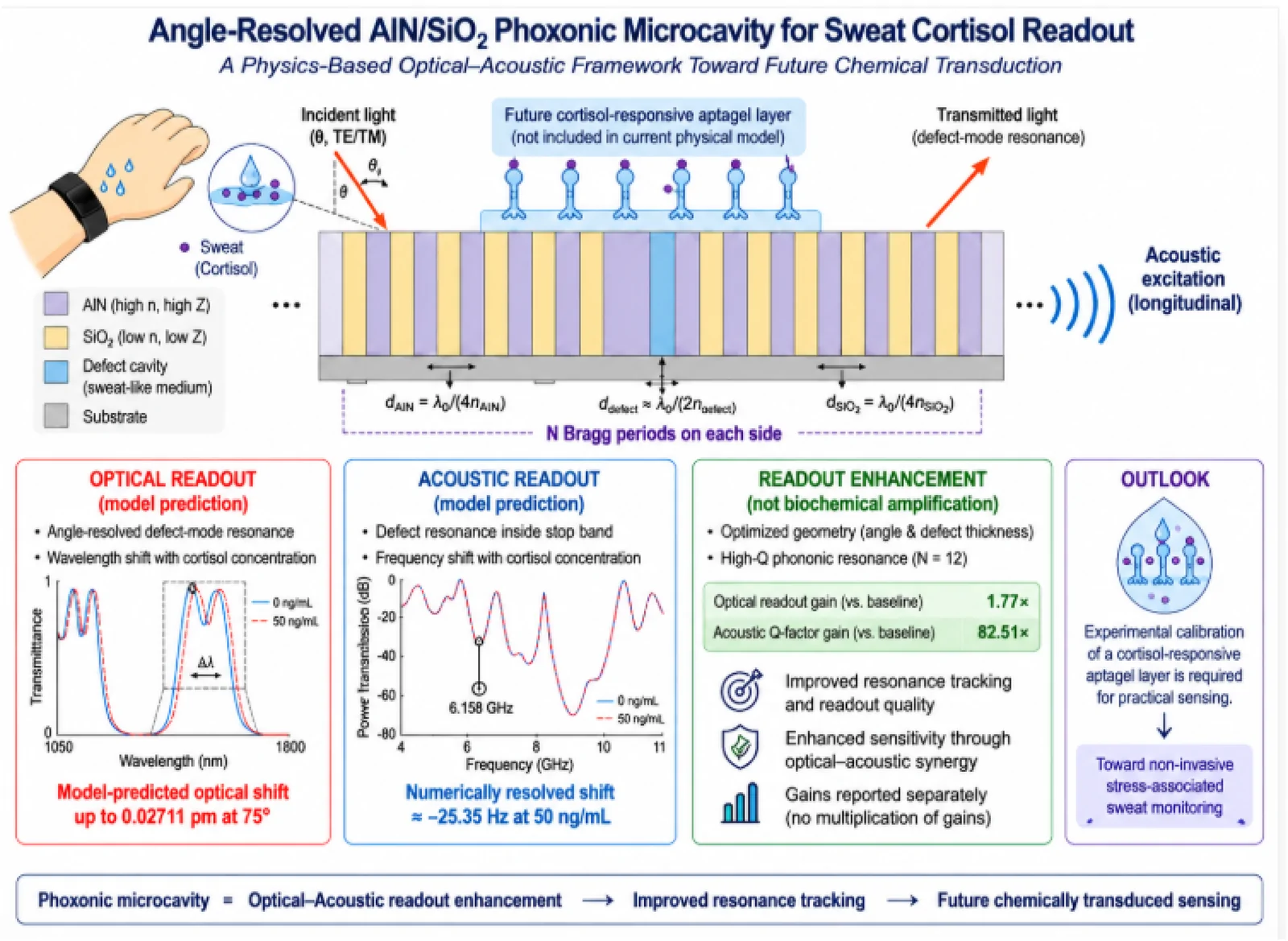
Conceptual design of an angle-resolved AlN/SiO_2_ phoxonic microcavity enabling enhanced optical–acoustic readout of sweat cortisol-related perturbations toward future receptor-mediated chemical transduction.

## Overview of the numerical framework and physical response limits

The numerical results were analyzed to establish the individual contributions of the optical and acoustic subsystems in the proposed phoxonic cavity framework. The analysis was intentionally performed by separating direct biochemical perturbation, optical resonance response, and acoustic confinement engineering. This approach avoids artificial enhancement of the sensing performance and allows each physical mechanism to be evaluated according to the implemented model.

The biochemical perturbation analysis indicates that the direct bulk effect of cortisol on the optical refractive index is intrinsically small. At a concentration of 50.0 ng mL^−1^, the calculated refractive-index perturbation is 9.802 × 10^−9^ RIU. Therefore, the optical response must be interpreted as a resonance-tracking problem rather than as a simple bulk-index detection mechanism. The calculated unloaded liquid-cavity acoustic response gives a frequency shift of −42.343 Hz at the same concentration, further demonstrating that direct bulk perturbation alone produces a weak acoustic response.

### Biochemical occupancy context and direct physical perturbation

The three panels in Fig. 2 provide an important physical separation between biochemical interaction and direct photonic/acoustic perturbation. The upper panel shows the increase of the Langmuir binding context with cortisol concentration, confirming that the biochemical interaction variable changes systematically with concentration. However, the lower panels demonstrate that the corresponding direct optical and acoustic material-property perturbations remain small within the implemented model. This comparison is important because it prevents interpreting biochemical occupancy as an artificial sensing enhancement. The result establishes the baseline limitation of direct bulk sensing and explains why cavity engineering and resonance-based readout are required in the proposed framework.

**Fig. 2:**
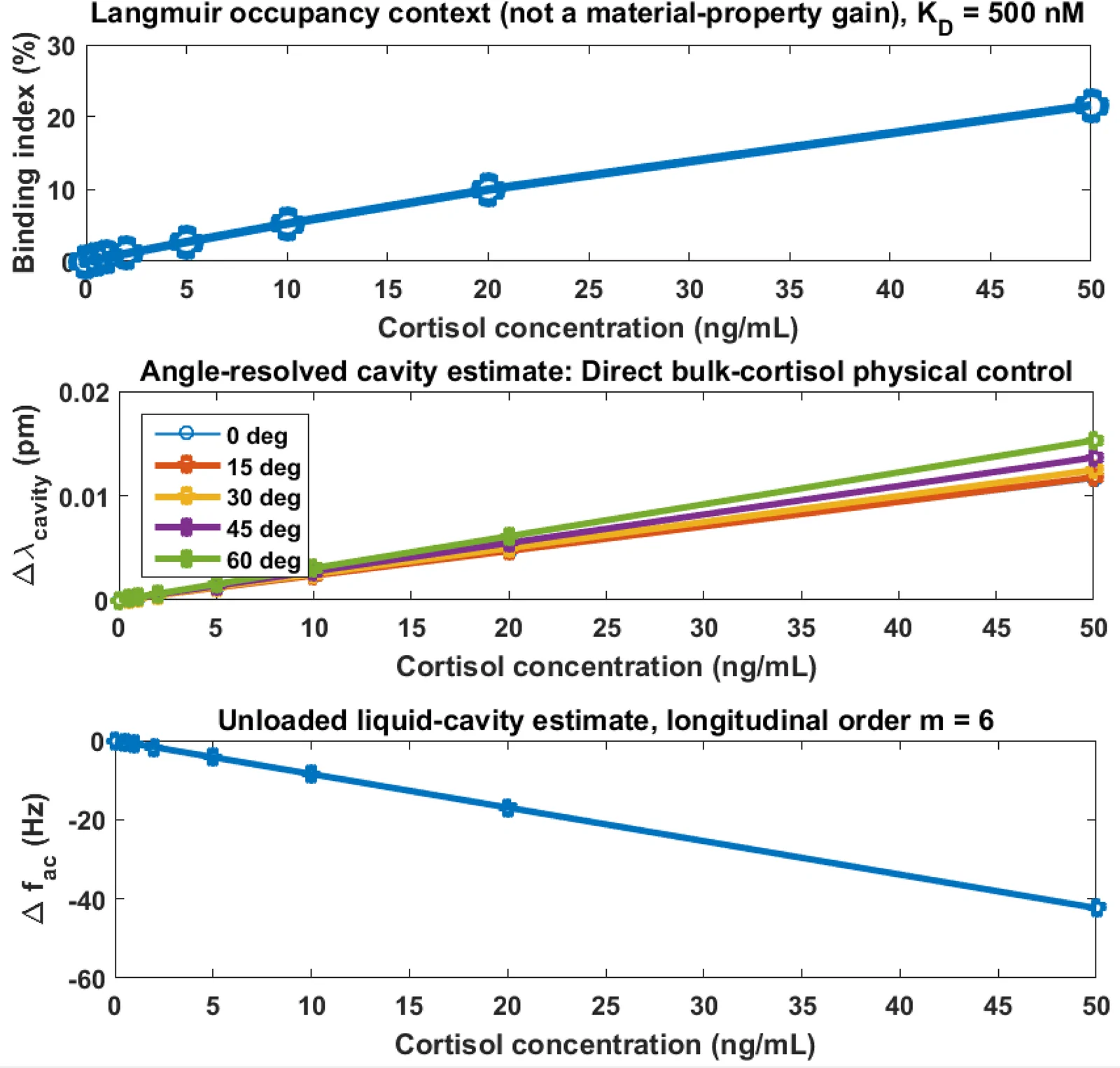
Langmuir occupancy context and direct bulk optical and acoustic perturbation limits for the investigated cortisol concentration range.

The behavior presented in Fig. 2 illustrates the distinction between biochemical occupancy and direct material-property perturbation. The Langmuir occupancy calculation increases with cortisol concentration, providing a biochemical context for receptor-mediated interaction. However, this occupancy curve is not interpreted as an additional material-property gain in the optical or acoustic model. Instead, the direct physical perturbation is evaluated independently.

This separation is essential because the calculated bulk perturbation remains limited. The result suggests that future experimental implementations would require receptor-mediated coupling or aptamer-based amplification mechanisms to convert biochemical binding into a larger effective perturbation. Within the current numerical framework, Fig. 2 establishes the physical baseline against which the cavity enhancement mechanisms are evaluated.

### Optical parameter screening and full-TMM verification

Fig. 3 illustrates the role of the preliminary parameter-screening step. The color distribution demonstrates how the estimated optical response changes with incidence angle and defect-thickness factor. The selected point at 75° incidence and a defect factor of 1.60 is used as a candidate geometry for subsequent full-TMM verification. The scientific importance of this map is not to provide an independent optimization result, but to visualize the parameter-dependent behavior and reduce the search space before the rigorous electromagnetic calculation.

**Fig. 3:**
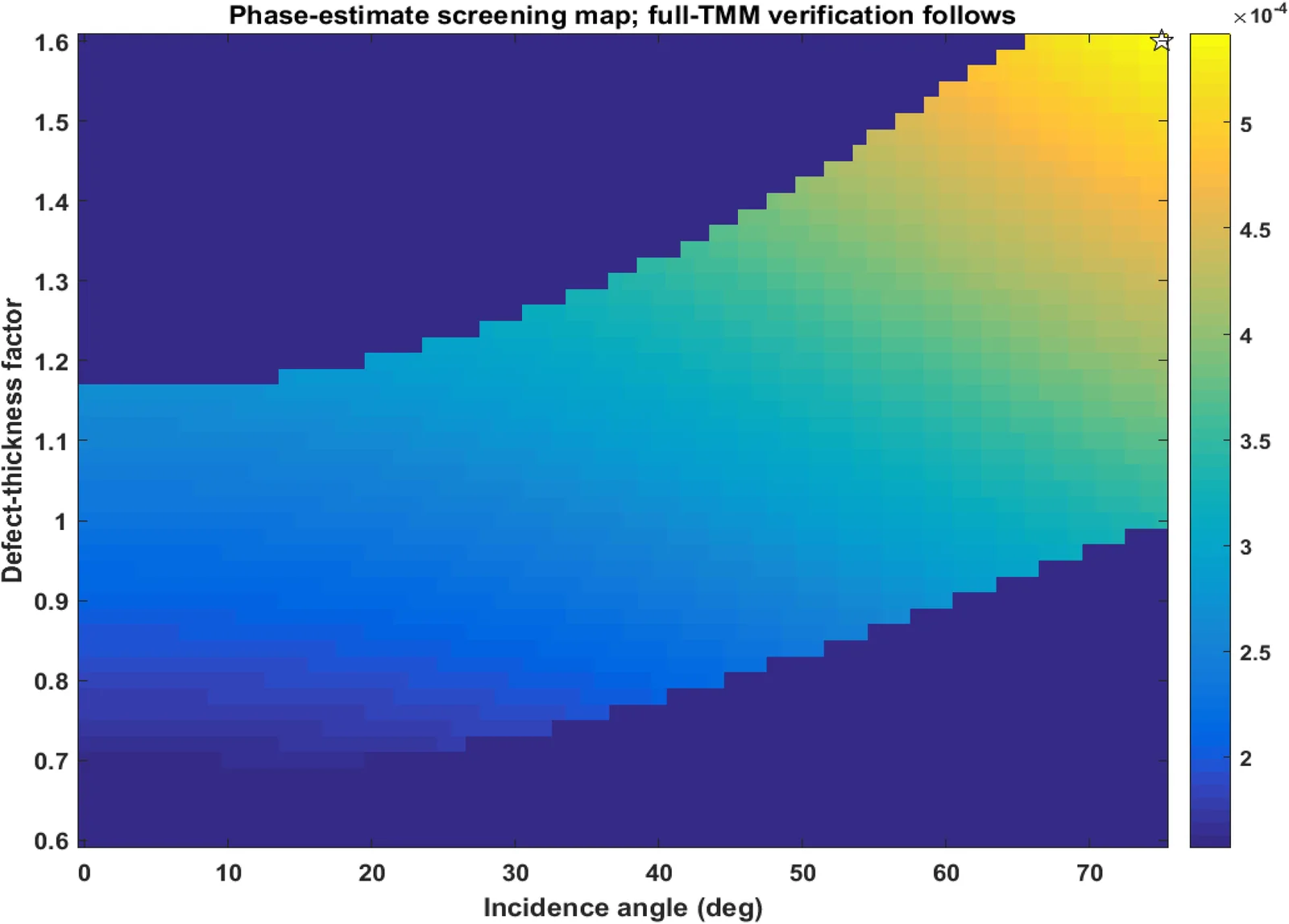
Phase-estimate screening map showing the candidate optical geometry before complete transfer-matrix verification.

The phase-estimate screening map in Fig. 2 presents the dependence of the estimated optical response on incidence angle and defect-thickness factor. The screened region identifies a candidate configuration at an incidence angle of 75° and a defect factor of 1.60. Importantly, this step is used only for parameter selection and not as a replacement for the complete electromagnetic calculation.

The physical significance of this result is that the parameter space can be narrowed before performing the computationally more demanding full-TMM calculation. The selected configuration is subsequently evaluated using the complete optical transfer matrix method to ensure that the reported response corresponds to the actual multilayer optical structure.

Fig. 4 represents the complete verification stage after the screening procedure. The almost coincident resonance curves before and after perturbation indicate that the direct refractive-index contribution produces an extremely small spectral displacement. The fitted shift therefore quantifies the intrinsic optical response of the structure under the investigated perturbation. Rather than indicating insufficient cavity performance, this result identifies the need for additional experimentally realistic coupling mechanisms, such as receptor-mediated interactions, in future implementations.

**Fig. 4:**
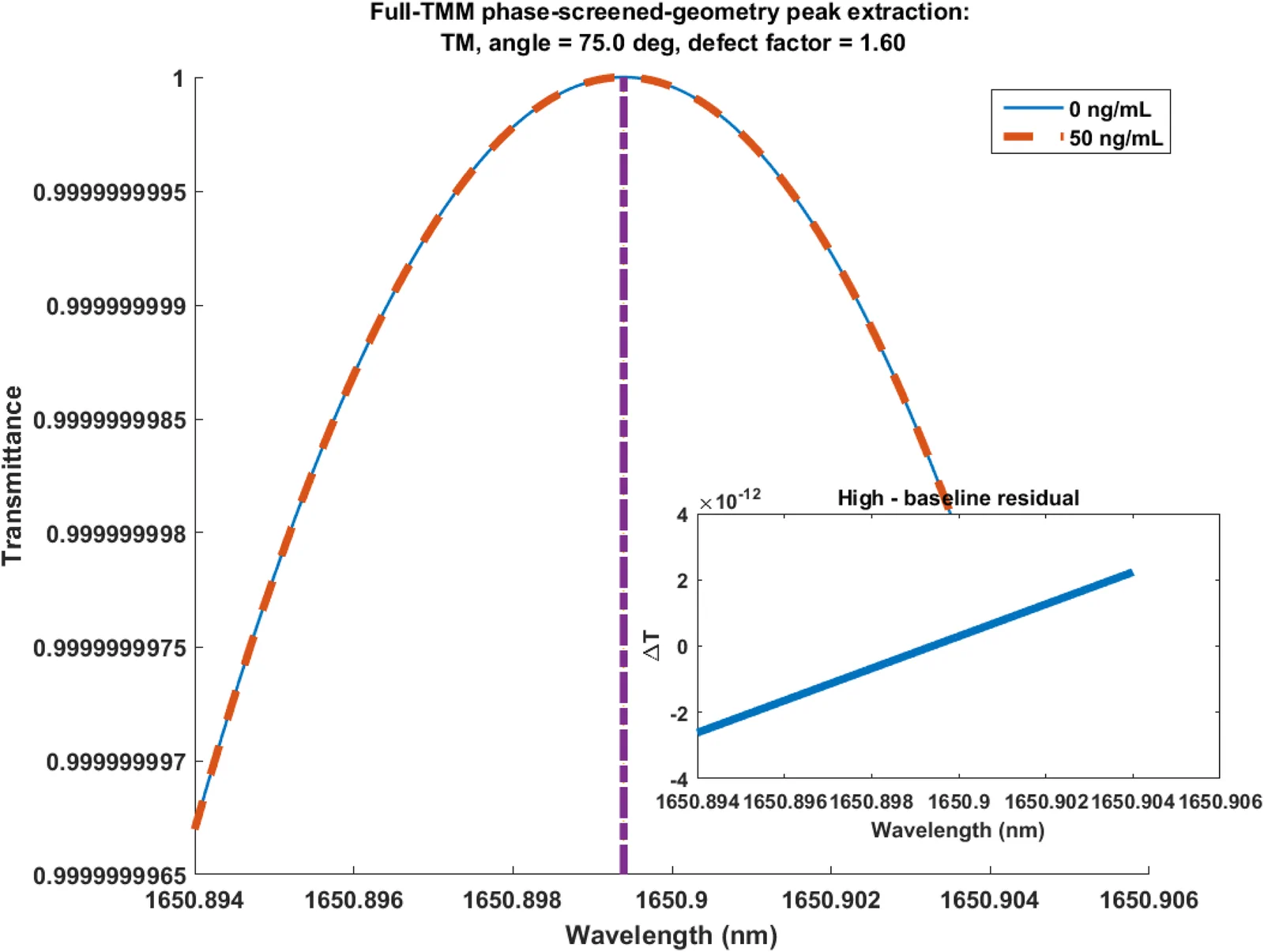
Full-TMM phase-screened peak extraction at the optimized TM condition (75° incidence, defect factor = 1.60), with the inset showing the magnified wavelength residual between the 0 and 50 ng mL^−1^ cases.

The response shown in Fig. 4 confirms the full-TMM evaluation of the selected geometry. The calculated TM fitted shift is 0.002 163 686 pm with a successful convergence flag. The nearly overlapping spectral responses before and after perturbation demonstrate the extremely small magnitude of the direct optical effect predicted by the model. Rather than indicating failure, this result defines the sensitivity requirement that must be addressed by future experimental coupling strategies.

### Comparative optical geometry analysis

The comparison presented in Fig. 5 provides a controlled evaluation of different optical geometries. The baseline configuration acts as the reference, while the angle-only and thickness-only cases reveal the isolated contribution of each parameter. The screened candidate combines the selected geometrical parameters and produces a favorable response among the tested configurations. The figure therefore demonstrates how geometry influences resonance sensitivity while maintaining the distinction between numerical improvement and global optimization.

**Fig. 5:**
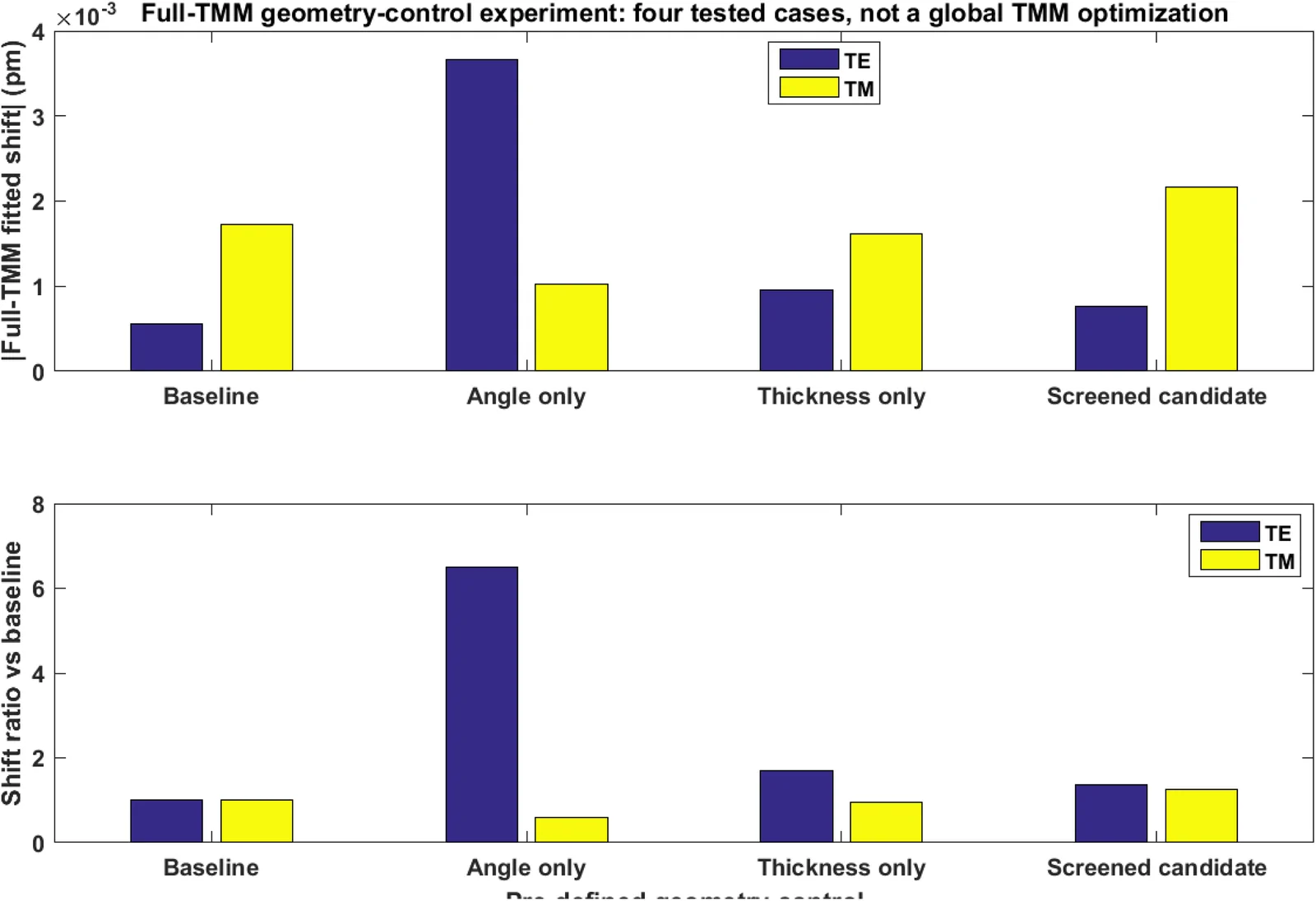
Full-TMM comparison of baseline, angle-only, thickness-only, and phase-screened optical geometries.

The comparison in Fig. 5 evaluates four predefined optical configurations. The baseline case provides the reference response, while the angle-only and thickness-only cases demonstrate the independent influence of each geometrical modification. The screened candidate combines the selected parameters obtained from the preliminary screening.

For TM polarization, the screened candidate produces a 1.2529-fold shift ratio relative to the baseline tested geometry. This improvement represents a comparative enhancement among the investigated cases only and should not be considered a global optimization result. The figure therefore demonstrates parameter-dependent behavior rather than claiming an absolute optimum.

#### Acoustic band structure and defect resonance formation

Fig. 6 connects the acoustic band analysis with the defect resonance calculation. The Bloch analysis identifies the frequency region where the acoustic structure supports the selected band behavior, while the full-stack TMM calculation confirms the defect-mode resonance inside this region. This agreement between band prediction and resonance extraction validates the acoustic design procedure and provides the basis for evaluating confinement-related parameters.

**Fig. 6:**
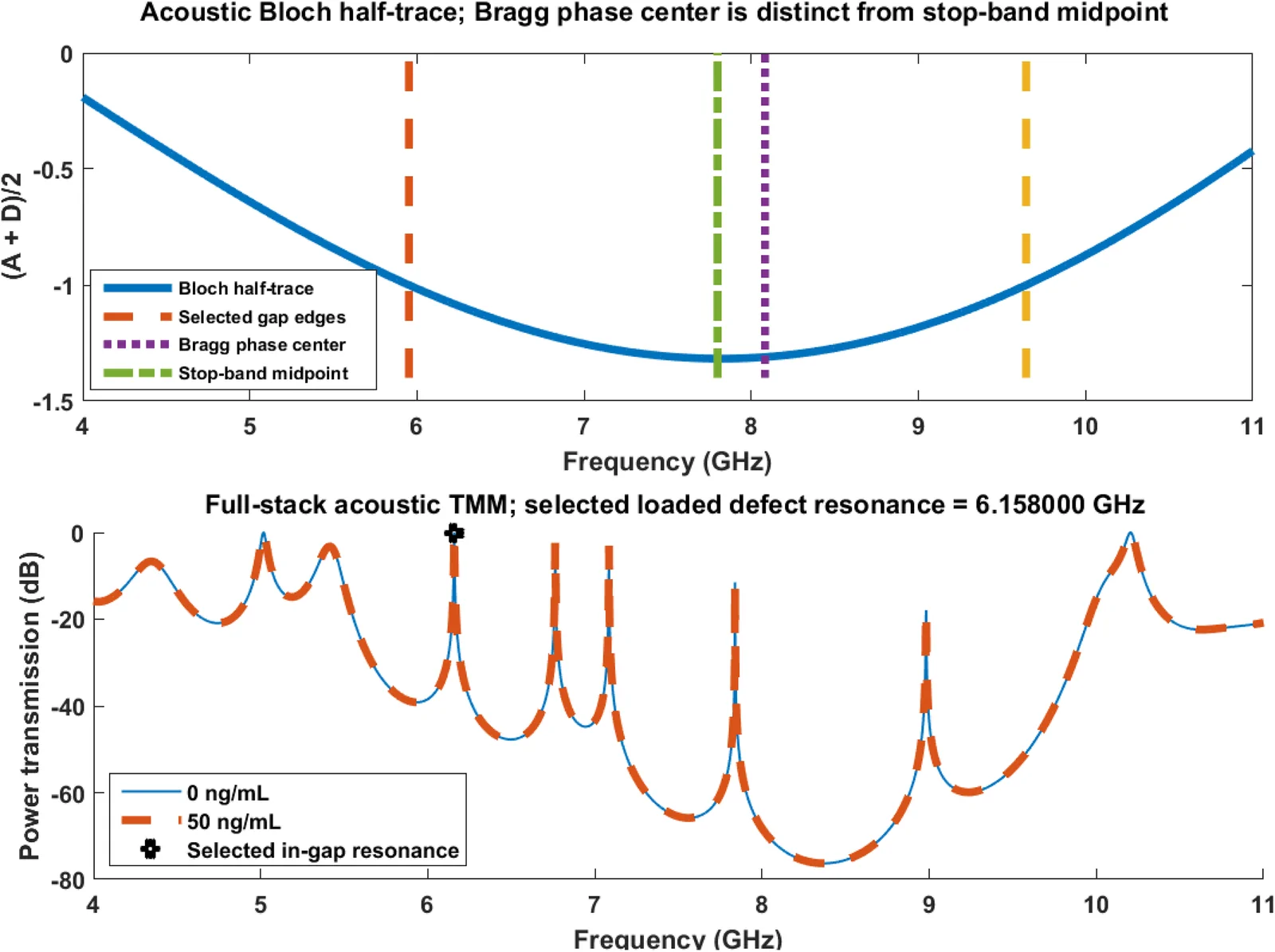
Acoustic Bloch analysis and full-stack TMM defect resonance identification.

The acoustic response presented in Fig. 6 demonstrates the formation of the defect resonance within the calculated stop-band region. The Bloch half-trace analysis identifies the selected acoustic band structure, while the full-stack acoustic TMM calculation locates the loaded defect resonance at 6.158 000 GHz.

The defect resonance provides the basis for evaluating acoustic confinement. Unlike the direct biochemical perturbation, the acoustic resonance position and linewidth are strongly affected by cavity design parameters, allowing systematic optimization of resonance quality.

#### Acoustic quality-factor optimization and numerical validation

The trends in Fig. 7 reveal the effect of increasing Bragg mirror periods on the simulated acoustic cavity performance. The increase in ideal-lossless *Q* and the reduction in FWHM indicate stronger numerical confinement as the mirror structure becomes more extended. At the same time, the figure highlights the inherent design trade-off because narrower resonances may require more demanding fabrication control. Therefore, the selected configuration represents a balance between numerical resonance quality and practical design considerations.

**Fig. 7:**
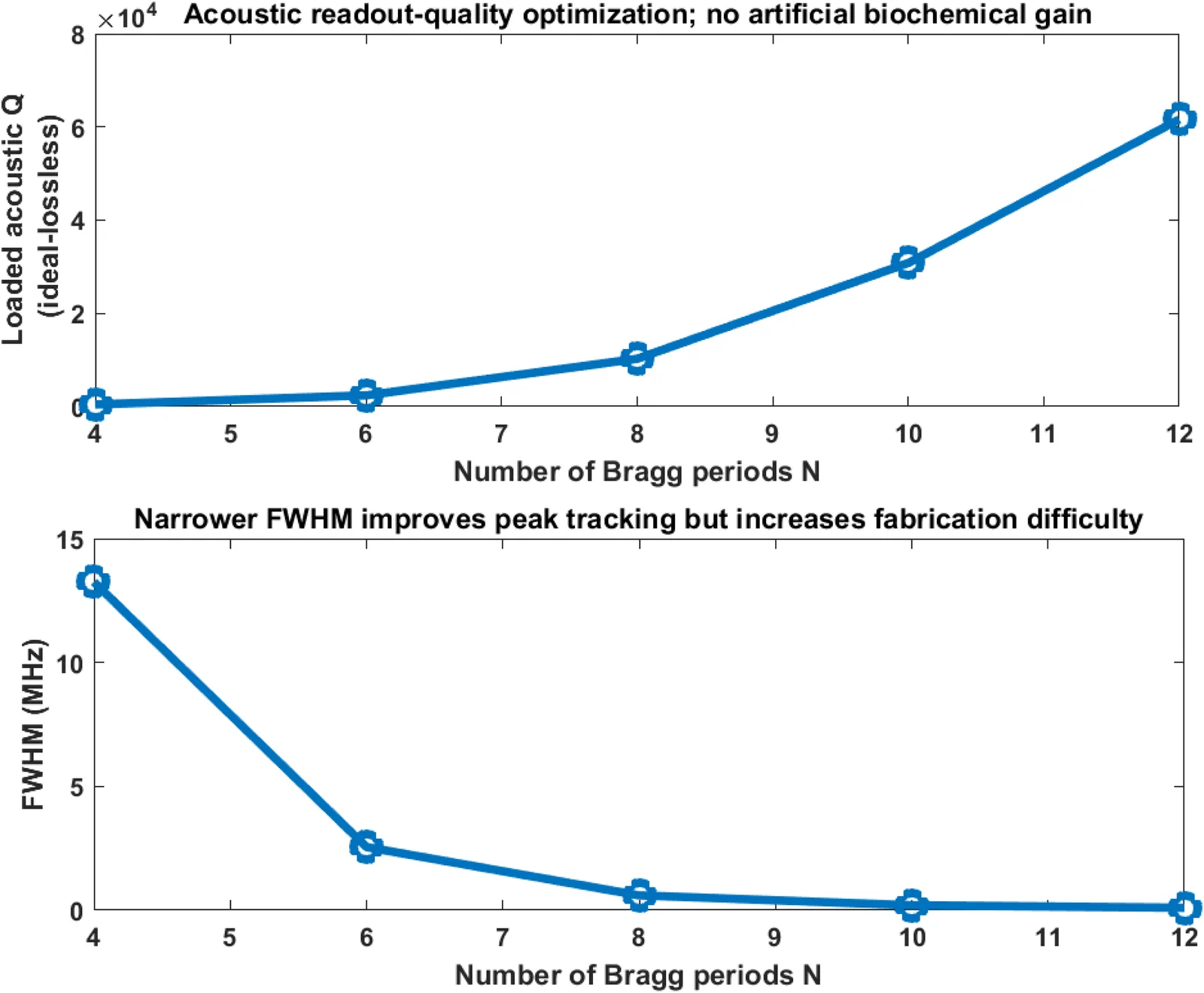
Dependence of ideal-lossless acoustic quality factor and linewidth on the number of Bragg mirror periods.

Fig. 7 demonstrates the influence of the number of Bragg periods on acoustic confinement. Increasing the mirror count produces higher ideal-lossless *Q* values and narrower FWHM values. The screening identifies *N* = 12 as the best investigated configuration with an ideal-lossless *Q* of 61 616.00 and FWHM of 0.100 000 MHz.

The observed trend is consistent with improved acoustic confinement in the simulated structure. However, these values represent ideal numerical conditions and should not be interpreted as experimentally achieved quality factors.

Fig. 8 provides the final numerical validation of the selected acoustic configuration. The convergence behavior with finer frequency scanning demonstrates that the extracted resonance parameters are stable with respect to numerical resolution. The simultaneous stabilization of *Q* and interpolated FWHM supports the reliability of the numerical extraction procedure and confirms that the reported acoustic performance originates from the modeled structure rather than from insufficient frequency sampling.

**Fig. 8:**
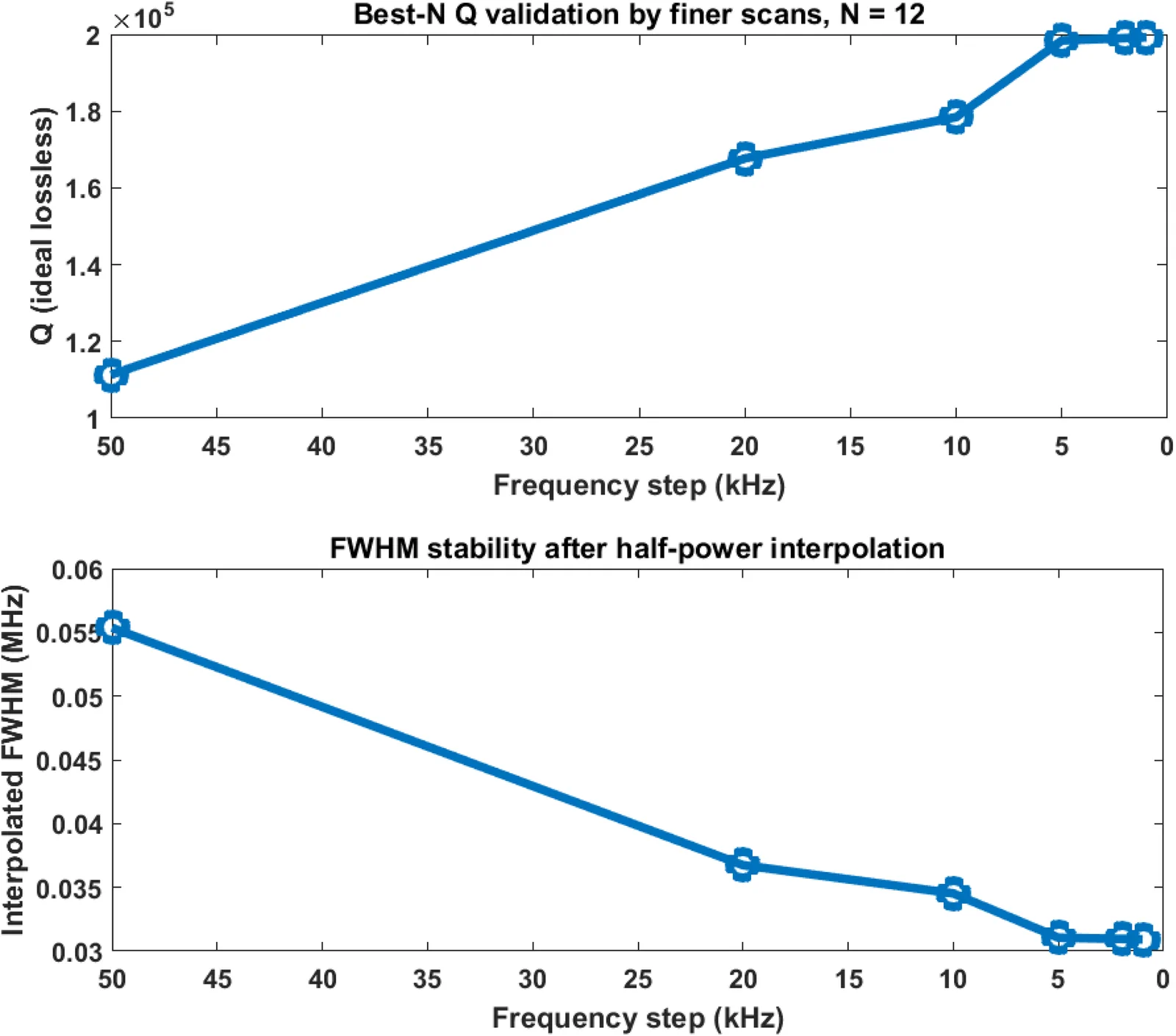
Fine-frequency-scan validation of the selected acoustic configuration and interpolation stability.

The convergence assessment in Fig. 8 confirms numerical stability of the selected acoustic configuration. At the finest frequency step, the calculated ideal-lossless *Q* reaches 199 263.12 with an interpolated FWHM of 0.030 922 MHz and a converged flag. This validation confirms computational consistency of the numerical procedure.

The acoustic *Q*-factor enhancement is reported only as a resonance-confinement metric, while the optical geometry improvement is treated independently as an optical readout parameter.

#### Physical interpretation and application relevance

Together, Fig. 2–8 demonstrate the different roles of biochemical interaction, optical resonance tracking, and acoustic cavity engineering. The optical subsystem provides a resonance-based interrogation framework, whereas the acoustic subsystem enables strong control over confinement through Bragg mirror design. The highest simulated acoustic performance results from increasing the mirror periods, while the optical response remains limited by the direct bulk perturbation.

Therefore, the proposed framework should be considered a proof-of-concept numerical platform for future receptor-mediated phoxonic sensing studies. Experimental validation would require incorporation of binding kinetics, measured optical and acoustic losses, instrument resolution, and resonance fitting uncertainty. Accordingly, the experimentally achievable detection limit will depend on the combined contribution of optical/acoustic instrument noise, resonance-fitting precision, and environmental stability, which cannot be quantified from numerical simulations alone.

## Model limitations

The current analysis is based on transfer-matrix calculations under defined numerical assumptions. The reported *Q* values correspond to ideal-lossless simulations and do not represent experimental measurements. In addition, the biochemical model describes direct perturbation and occupancy context rather than a complete receptor-mediated sensing experiment.

These limitations define the scope of the present work but do not prevent the model from serving as a useful framework for evaluating parameter selection, resonance engineering, and future experimental designs.

The present numerical framework does not represent a complete cortisol sensing experiment because receptor-mediated biochemical amplification and experimentally measured transduction efficiency are not included. Therefore, the reported optical and acoustic enhancements should be interpreted as improvements in resonance readout capability rather than direct analyte amplification. Future experimental implementation will require integration and calibration of a cortisol-responsive receptor layer to establish quantitative sensing performance.

### Quantitative summary of the investigated cases

To further clarify the relationship between the simulated parameters and the obtained responses, the main numerical outcomes are summarized in Table 1. The table is not intended as an additional optimization step, but as a compact representation of the values already extracted from the implemented numerical framework. The optical and acoustic metrics are kept separated because they describe different physical mechanisms.

**Table 1:** Summary of key optical and acoustic simulation results and their physical interpretation

Analysis component	Investigated parameter/case	Reported numerical outcome	Scientific interpretation
Direct optical perturbation	Cortisol concentration = 50 ng mL^−1^	Δ*n* = 9.802 × 10^−9^ RIU	Indicates that direct bulk refractive-index modification is small and resonance tracking is required
Optical geometry screening	75° Incidence angle, defect factor = 1.60	Selected screened configuration	Provides a candidate geometry before complete full-TMM verification
Optical full-TMM verification	Phase-screened geometry	TM fitted shift = 0.002 163 686 pm	Confirms the small direct optical response predicted by the model
Acoustic resonance	Loaded defect configuration	Defect resonance = 6.158 000 GHz	Demonstrates defect-mode formation inside the calculated acoustic structure
Acoustic validation	*N* = 12, Ideal-lossless condition	*Q* = 199 263.12; Interpolated FWHM = 0.030 922 MHz	Represents numerical confinement quality under ideal simulated conditions

### Extended interpretation of the comparative results

The comparative analysis should be interpreted as a sequence of controlled numerical experiments. The baseline configurations provide the reference state, while modified geometries isolate the effect of individual parameters. This approach allows the contribution of incidence angle, defect thickness, and Bragg mirror periodicity to be evaluated independently before considering their combined effect.

The optical results demonstrate that geometric tuning can modify the resonance response, but the magnitude of the direct biochemical perturbation remains the limiting factor within the current model. Accordingly, the calculated enhancement should be understood as improved resonance discrimination among the tested geometries rather than as an increase in the intrinsic biochemical interaction.

The acoustic results provide a complementary perspective. The increase in Bragg periods improves the simulated confinement and reduces the linewidth under ideal-lossless assumptions. Therefore, the acoustic design parameter controls the numerical resonance quality, whereas the biochemical response itself is not artificially amplified in the implemented calculations.

## Conclusion

This study presented a numerically validated AlN/SiO_2_ phoxonic microcavity for angle-resolved optical–acoustic readout of cortisol-containing sweat-like media. The model intentionally separated the weak direct bulk response of cortisol from the readout enhancement provided by the phoxonic architecture. The results confirmed that free cortisol in an aqueous defect produces only minute optical and acoustic perturbations, indicating that direct bulk sensing alone is insufficient for practical operation. However, optical geometry optimization improved resonance tracking, while acoustic mirror-count optimization produced a highly confined defect resonance with a high numerical quality factor under the investigated conditions. These findings demonstrate that the proposed phoxonic microcavity provides a readout-quality-enhanced framework for weak biochemical perturbations.

The reported improvement should be interpreted as enhanced optical–acoustic resonance tracking rather than biochemical amplification. Practical cortisol sensing will require an experimentally calibrated cortisol-responsive aptagel or related receptor-mediated transduction layer capable of converting molecular binding events into measurable defect-property variations. Within these limitations, the proposed AlN/SiO_2_ platform provides a physically consistent proof-of-concept framework toward future chemically transduced sweat-cortisol monitoring and may support the development of non-invasive stress-associated physiological monitoring systems.

## Ethical statement

Ethics approval was not required for this study because it did not involve human participants, human tissues, biological samples, animal experiments, or clinical data. The work is entirely based on numerical simulation and computational modeling.

## Author contributions

The manuscript was prepared by the author, who carried out the conceptualization, methodology, numerical modeling, formal analysis, investigation, visualization, original draft preparation, and manuscript revision. The author has read and approved the final version of the manuscript.

## Conflicts of interest

The author carried out all aspects of the study, including conceptualization, modelling, analysis, visualization, and manuscript preparation.

## Data Availability

The data generated and analyzed during the current study are available from the corresponding author upon reasonable request.
